# Policies, recommendations and training to respond to patient microaggressions and hate speech aimed at healthcare professionals: A systematic review

**DOI:** 10.1192/j.eurpsy.2021.1029

**Published:** 2021-08-13

**Authors:** D. Wittkower, J. Bryan, A. Asghar-Ali

**Affiliations:** 1 Psychology, university of Houston, Houston, United States of America; 2 Mirecc, VA South Central Mental Illness Research, Education and Clinical Center (MIRECC), Houston, United States of America; 3 Mirecc, Micheal E DeBakey VA, Houston, United States of America; 4 Mirecc, VA South Central Mental Illness Research, Education and Clinical Center, Houston, United States of America; 5 Menninger Department Of Psychiatry And Behavioral Sciences, Baylor College of Medicine, Houston, United States of America

**Keywords:** Patient discrimination and microaggressions and hate speech and training

## Abstract

**Introduction:**

Patient microaggressions and hate speech affect practitioners in all fields of healthcare. In some facilities, 100% of healthcare workers report experiencing harassment and hate speech, with the aggressors most frequently being patients. To date, there has been no systematic review of policies, recommendations and trainings on patient microaggressions and hate speech against healthcare professionals.

**Objectives:**

A systematic review was conducted to identify recommendations and solutions for healthcare professionals on responding to patient microaggressions and hate speech. Additionally, websites of major healthcare professional organizations and the 6 largest healthcare systems were checked for policy statements related to discrimination by patients towards healthcare providers.

**Methods:**

A literature search of PubMed, PsycINFO, Medline, ERIC and MedEdPORTAL. Articles that contained recommendations and trainings for responding to microaggressions and hate speech were retained. 13 Leading professional organizations and 6 healthcare systems were checked for policies on discrimination by patients.

**Results:**

Our review identified 27 studies providing recommendations and trainings for healthcare professionals to address patient hate speech and microaggressions. Three professional organizations but no healthcare systems had policies on discrimination by patients.

**Conclusions:**

Seven trainings that equip providers with tools to address patient microaggressions and hate speech were identified. Trainings included the ERASE framework; Stop, talk, and roll; interrupting microaggressions; and the OWTFD tool. Nineteen studies outlined recommendations for healthcare professionals and systems on how to respond to patient offenses. Professional organizations and healthcare systems need to create policies to support healthcare professionals who face microaggressions and hate speech.

